# Association of the COVID-19 Pandemic With Medical School Diversity Pathway Programs

**DOI:** 10.1001/jamanetworkopen.2022.29086

**Published:** 2022-08-29

**Authors:** Sonal Batra, Julie Orban, Shalini Raichur, Nicholas Jennings, Charmi Trivedi, Nehal Naik, Colleen Bogucki, Yolanda Haywood

**Affiliations:** 1 Department of Emergency Medicine, School of Medicine and Health Sciences, The George Washington University, Washington, DC; 2 Department of Health Policy and Management, Milken Institute School of Public Health, The George Washington University, Washington, DC; 3 Department of Health Policy and Management, Milken Institute School of Public Health, The George Washington University, Washington, DC; 4 NORC at the University of Chicago, Bethesda, Maryland; 5 School of Medicine and Health Sciences, The George Washington University, Washington, DC; 6 Department of Emergency Medicine, School of Medicine and Health Sciences, The George Washington University, Washington, DC

## Abstract

**Importance:**

Medical school pathway programs are a strategy to increase the diversity of the physician workforce. The COVID-19 pandemic may have negatively affected pathway programs, further challenging efforts to increase diversity.

**Objectives:**

To describe the changes in medical school pathway programs during the COVID-19 pandemic and identify methods for sustaining and supporting these programs during and after the pandemic.

**Design, Setting, and Participants:**

A survey study using an exploratory sequential mixed-method design was conducted from January 4 to August 3, 2021. Semistructured interviews with a sample of medical school pathway program administrators and academic leaders of US allopathic and osteopathic medical school diversity pathway programs identified themes and patterns of change to pathway programs since the onset of the pandemic compared with previous years. These themes were used to develop a survey that was sent to medical schools to assess the association between COVID-19 and their programs.

**Main Outcomes and Measures:**

The association between the COVID-19 pandemic and medical school diversity pathway programs was explored using interview and survey data assessing respondent characteristics; changes in the scope, size, and funding of programs in 2020 compared with previous years; and respondents’ perceptions of future needs for pathway programs.

**Results:**

Twelve program administrators and academic leaders were interviewed. Interviews revealed challenges and benefits of virtual programming in engaging and reaching students and speakers, the value of community partnerships to sustaining programming, and the importance of psychosocial support to mediating students’ mental health challenges due to COVID-19 and remote learning. Of 198 schools surveyed in the quantitative phase, 112 responded (56.6%), 106 (94.6%) of which had been sponsoring or assisting with pathway programs during the COVID-19 pandemic. Forty-two respondents (39.6%) had reduced pathway programs since the onset of the COVID-19 pandemic compared with the previous year. Program cancellations were more likely to be noted in elementary school–aged (50.0% decrease in programming; *P* = .01) and middle school–aged (32.6%; *P* = .02) students compared with older groups.

**Conclusions and Relevance:**

In this survey study, schools indicated that pathway programs were disrupted by COVID-19. Ongoing and flexible supports may be needed to sustain these programs. These findings are timely given recent investments in equity-focused programs to diversify the health workforce.

## Key Points

**Question:**

Is the COVID-19 pandemic associated with changes in medical school diversity pathway programs?

**Findings:**

This survey study of 12 medical school pathway program administrators and 112 osteopathic and allopathic schools found a decrease in diversity pathway programming since the onset of the COVID-19 pandemic compared with the previous year. The participants reported that in-person experiences, including research and shadowing, and programs targeting elementary and middle school–aged students appeared to be the most affected.

**Meaning:**

The findings of this study suggest that diversity pathway programs were substantially disrupted by the COVID-19 pandemic; the long-term outcomes of these disruptions are unknown.

## Teaser

This survey study examines the status of medical school diversity pathway programs during the COVID-19 pandemic compared with previous years.

## Introduction

Medical school diversity pathway programs—also referred to as pipeline, enrichment, or preprofessional programs—are a set of interventions designed to increase the diversity of the physician workforce.^1^ These programs are required by the Liaison Committee on Medical Education^2^ and include components such as academic enrichment, admissions preparation, mentorship, financial support, psychosocial support, and career exposure opportunities. The Liaison Committee on Medical Education is recognized by the US Department of Education to accredit medical education programs leading to the MD degree. Programs must demonstrate compliance with standard 3.3 Diversity/Pipeline Programs and Partnerships (standards for effective years 2022-2023).^3,4^

A diverse physician workforce is associated with reduced health care disparities, increased access to health care services, and improved quality of care to patients.^5,6^ Studies also suggest positive patient-centered outcomes associated with patient-clinician concordance including patient care satisfaction,^7^ continuity of care,^8^ and trust,^9^ especially for the most vulnerable individuals.^10^ Student body diversity produces educational benefits such as racial and cultural awareness to care for racial and ethnic minority populations and strong attitudes toward equitable access to care.^11^ A physician workforce that is not only adequate in size and specialty mix but that also reflects the increasing racial and ethnic diversity of the nation is a necessary element to the advancement of health equity in the US.^6^ Nonetheless, despite efforts by medical education leaders,^12,13^ Black, Latinx, and Native American people constitute only 11% of US physicians^14^ although they make up 32% of the total US population.^15^ As defined by the Association of American Medical Colleges, *underrepresented in medicine* means racial and ethnic populations that are underrepresented in the medical profession relative to their numbers in the general population.^16^ Creating a physician workforce that is in closer alignment with the general population is a goal of these pathway programs.

In 2020, the COVID-19 pandemic created a mass disruption for schools across the country, with educational organizations ranging from kindergarten through high school, undergraduate institutions, and medical schools adjusting from in-person education to online learning. This disruption exacerbated preexisting educational and resource disparities, with grade schools serving students of lower economic status and marginalized racial and ethnic groups less able to meet technology needs, provide both physical and virtual access to teachers regularly, and reach all students.^17,18,19,20^ Many medical schools transitioned core educational activities to virtual schooling, but certain extracurricular and noncore activities, including pathway programs, may have been scaled down or canceled during the uncertain transition to the virtual environment.

The aim of this study was to investigate the association between diversity pathway programs at medical schools, specifically those aimed at increasing representation of racial and ethnic minority populations in the medical profession, and the COVID-19 pandemic. Although the long-term outcome of COVID-19 regarding the diversity of the physician workforce will take decades to elucidate, this analysis identifies (1) changes in diversity pathway programs since the onset of the COVID-19 pandemic, (2) how these changes may have been variably associated with different types of medical schools, and (3) methods for supporting and sustaining these programs during and after the pandemic.

## Methods

This study including semistructured interviews and a survey using an exploratory sequential design^21^ was conducted from January 4 to August 3, 2021. We interviewed a sample of 12 medical school diversity pathway program administrators and academic leaders to identify themes and patterns of change to diversity pathway programs since the onset of the COVID-19 pandemic compared with previous years. These themes and patterns were used to inform a survey that was administered to all US medical schools to assess the association between COVID-19 and their programs. We followed the American Association for Public Opinion Research (AAPOR) reporting guideline for survey studies. The George Washington University Institutional Review Board approved the study. Verbal consent was obtained from interview participants and a waiver of documentation of consent was approved for survey participants. No financial incentives were provided for participation.

### Semistructured Interviews

We conducted 1-hour interviews by electronic conference platform (Zoom Video Communications Inc)^22^ with program administrators and academic leaders between January 8 and April 9, 2021. We identified and recruited participants using a multisampling strategy. Participants were selected from the roster files of the Association of American Medical Colleges for allopathic schools and the American Association of Colleges of Osteopathic Medicine for osteopathic schools using purposive sampling—schools of varying sizes, institutional affiliations, ownership (private vs public), types of populations served (urban vs rural), and geographic locations. We contacted individuals who were listed on institutional websites as running their school’s pathway programs by email or telephone. If there was not an individual specifically listed, we contacted the assistant/associate dean for diversity, equity, and inclusion or someone in a similar role. We developed a list of other potential participants through professional contacts, the literature, and snowball referrals.

One of us (S.B.) led the interviews with additional team members joining (J.O., S.R., C.T.) based on availability. A semistructured interview guide developed from a review of the literature (hereafter referred to as *guide*) was used. The guide included questions about operational characteristics, program goals and measures, the outcomes of the COVID-19 pandemic, and recommendations (eAppendix 1 in the Supplement).

We used a rapid thematic content approach^23^ to identify and iteratively analyze patterns in the data. Interviews were videorecorded, transcribed with field notes (S.R., C.T.), and coded (J.O., S.R.) inductively (derived from the interviews, discussions, and notes) into a matrix of themes.^24^ Recorded video interviews were reviewed as needed for clarification of the transcribed text. Findings were validated through triangulation among the research team.

### Survey

We developed a 13-question survey informed by themes revealed from the interviews, a literature review, and the social mission metrics framework—a formalized method to assess health professions school social mission values, programs, and activities that include pathway programs.^25^ The survey was tested with 3 medical school pathway program leaders to assess clarity and comprehensiveness and reviewed by an expert survey scientist to ensure best practice in survey methods. Survey questions assessed respondent characteristics, changes in pathway program scope, size, and funding in 2020 compared with previous years, and respondents’ perceptions of future needs for pathway programs (eAppendix 2 in the Supplement). The survey included 2 open-ended questions to elicit unexpected outcomes associated with COVID-19 and medical school pathway programs and additional comments.

All allopathic (n = 155) and osteopathic (n = 42) medical school main campuses were invited to complete the electronic survey. We also contacted an additional osteopathic branch campus in a different state from its main campus for a total of 198 schools. A comprehensive list of US medical schools was obtained using the Association of American Medical Colleges and American Association of Colleges of Osteopathic Medicine roster files. The survey was administered through an online survey platform (Qualtrics) between June 10 and August 3, 2021. The survey was directed to the leadership of the offices of diversity, equity, and inclusion, or similar roles at each school. A list of recipients’ email addresses was obtained via online searches of the school’s diversity office website, faculty directory, or pathway program contact listing. Participants received an initial email with a cover letter and the survey link. Nonrespondents received 10 reminder emails over the course of 8 weeks to encourage completion. Toward the end of the survey period, telephone numbers were retrieved via an online search similar to the email retrieval process for nonrespondents and calls were made to ensure receipt of the survey and confirm contact information.

### Statistical Analysis

Analysis was conducted using χ^2^ and Fisher exact tests to compare characteristics of participating and nonparticipating schools as well as assess the association between school characteristics and their survey responses. Two-tailed paired *t* tests were used to examine the changes in programming from before the pandemic to during the pandemic. Statistical significance was set at a threshold of *P* = .05.The school characteristics analyzed included school type (allopathic or osteopathic), region, ownership status (public or private), Carnegie Classification, status as community-based schools, and status as racial and ethnic minority group–serving institutions (historically Black colleges and universities or Hispanic-serving institutions). Survey responses were analyzed by conducting descriptive analyses using Stata, version 17 (StataCorp Inc).

## Results

### Semistructured Interviews

Twelve of 29 invited individuals (41.4%) participated in interviews. A priori estimates of the number of interviews needed was 10 to 15; interview invitations ended once thematic saturation was reached. We identified 7 major themes from the interviews: (1) programmatic changes, (2) participant engagement, (3) technological barriers, (4) increased psychosocial support for students, (5) community partnerships, (6) financial factors, and (7) potential hybrid programming in the future. These thematic findings were supported by statements from the participants (Table 1).

**Table 1.** Thematic Findings and Supporting Statements from Semistructured Interviews, 2021

Themes and subthemes	Supporting statements
**Theme 1: programmatic changes**	
Distance learning/instruction	…So we had to make some plans, really, really quickly to move everything [to a] virtual format.
	We had to go to all virtual.
Program cancellation	So of our 5 camps, 3 of them had to be canceled.
**Theme 2: participant engagement**	
Decreased student engagement	And so they [students] don’t want to be engaged in a precollege program anymore. They’re kind of just trying to get through a semester of virtual school and not really focused on anything else.
	I have seen a change in the level of involvement, if you will…just generally speaking, that sense of community…
Spreading programmatic reach to new students and speakers	I think one of the incredible lessons we learned was the ability to really engage participants that would not have otherwise been able to attend in person. We were able to reach more by Zoom than we probably would have in terms of if you look at regional and everything else than we would have been in person.
	I’m able to reach out to alumni and speakers, you know, across country and bring them in, if you will.
**Theme 3: technological barriers**	
Varied access and resources	…Not everyone had the same type of computers, so we have some students who are working strictly from iPads. We had at some points [students] had to work from their phones…. We had a lot of tech issues…computer might have been freezing or they just didn’t have the software, so like Adobe…
	You know, some students not either having the bandwidth or the technology.
**Theme 4: increased psychosocial support for students**	
Mental health impacts	It really did hurt us a bit spring semester (when COVID hit) because a lot of our students just felt lost…really stressed… behind on their schoolwork, and…haven’t even thought about college.
	I would say our case manager has really done double time…I added another counselor…we’re doing a lot more coaching and counseling for personal and wellness…
**Theme 5: community partnerships**	
Value of partnerships for sustainability	So, I definitely agree that already having the trust and the relationship [of existing partnerships] helped there so that we could move quickly. It still meant like you’re working and doing all that, but you could work a little more nimble with that for sure.
	You’re never going to have success unless you have a strong community partnership…being in private universities, you have to find public sector partners and you need to define shared goals and figure out how you can work together to do more than you can on your own. So that public private capacity building, I find is really important…sustainability planning should start from the very beginning…
**Theme 6: financial factors**	
Changes in funding	So our grant funding did not decrease, fortunately, but what happened is a lot of our host institutions had to take funding decreases.
Institutional budget cuts and hiring freezes	My budget was cut. We have a hiring freeze.
	So we had to lay off and furlough people because of COVID-19…the main person in charge of this program has had her time reduced slightly because of furlough.
**Theme 7: potential hybrid programming in the future**	
Plans for the future	I think we will implement more virtual type programming and not require so much in-person.

### Survey

Of the 198 schools invited to participate, 112 completed the survey (56.6%) (Table 2).^26,27,28^ There were no significant differences in response rates across medical school characteristics.

**Table 2.** Characteristics of Schools in the Survey, 2021

Characteristic	No. (%)		
	Invited (n = 198)	Participating (n = 112)	Response rate, %
School type			
Allopathic (MD)	155 (78.3)	92 (82.1)	59.4
Osteopathic (DO)	43 (21.7)	20 (17.9)	46.5
Region^a^			
Midwest	42 (21.2)	24 (21.4)	57.1
Northeast	43 (21.7)	21 (18.8)	48.8
South	75 (37.9)	40 (35.7)	53.3
West	34 (17.2)	24 (21.4)	70.6
Puerto Rico	4 (2.0)	3 (2.7)	75.0
Ownership status			
Public	101 (51.0)	59 (52.7)	58.4
Private	97 (49.0)	53 (47.3)	54.6
Institution classification^b^^,^^c^			
Doctoral universities			
R1	78 (39.6)	46 (41.1)	59.0
R2	31 (15.7)	15 (13.4)	48.4
R3	12 (6.1)	5 (4.5)	41.7
Medical schools and centers	71 (36.0)	44 (39.3)	62.0
All others	5 (2.5)	2 (1.8)	40.0
Community-based status^d^			
Osteopathic	43 (21.7)	20 (17.9)	46.5
Allopathic			
Community based	34 (17.2)	25 (22.3)	73.5
Noncommunity based	121 (61.1)	67 (59.8)	55.4
Racial and ethnic minority–serving institutions			
Historically Black colleges and universities	3 (1.5)	1 (0.9)	33.3
Hispanic-serving institution	26 (13.1)	18 (16.1)	69.2
Total	198 (100)	112 (100)	56.6

Abbreviations: DO, doctor of osteopathic medicine; MD, doctor of medicine.

^a^ Region is based on Census regions and divisions of the US.^26^

^b^ Institutional classification is based on the Carnegie Classification of Institutions of Higher Education, 2021.^27^

^c^ Excludes 1 school of osteopathic medicine that did not have a Carnegie Classification.

^d^ Community-based status for allopathic schools is from the Association of American Medical Colleges Organizational Characteristics Database, 2021.^28^

### Association Between COVID-19 Pandemic and Programming

Of the 112 schools that responded to the survey, most schools were running at least 1 pathway program both before (108 [96.4%]) and during (106 [94.6%]) the pandemic. The survey included 1 exclusion question: “During the COVID-19 pandemic, has your school been sponsoring, running, or assisting with any pathway programs targeted to K-12 [kindergarten through high school] and/or undergraduate college students who are underrepresented in medicine aimed at encouraging or preparing them to train for careers in the health professions?” The survey ended for 6 respondents who answered no to the question, leaving 106 schools that completed the full survey. Forty-two of 106 respondents (39.6%) reported canceling some or all of their programs because of the COVID-19 pandemic.

Specific types of programmatic activities were variably affected by the COVID-19 pandemic (Table 3). Elementary and middle school programs were less common than high school and undergraduate programs before the pandemic, and also had significant decreases in programming during the pandemic (50.0% decrease for elementary schools; *P* = .01; 32.6% decrease for middle schools; *P* = .02) compared with high school (4.3% decrease; *P* = .22), undergraduate (2.2% decrease; *P* = .35), and postgraduate (4.9% decrease; *P* = .34) students. The most common program type offered was mentoring, which appeared to be least affected by the pandemic. Shadowing and internship opportunities were significantly decreased (56.5%; *P* < .001), as were research experiences, which decreased by 16.9% (*P* = .03). Conversely, the number of schools providing distance learning support doubled during this time. Still, distance learning remained one of the least common program types offered (21 of 106 [19.8%] schools before the pandemic; 42 of 106 [39.6%] schools since the onset of the pandemic).

**Table 3.** Programmatic Activities of Medical School Pathway Programs

Variable	No. (%)			*P* value^a^
	Before the pandemic (n = 106)	During the pandemic (n = 106)	% Change	
Educational level				
Elementary school	24 (22.6)	12 (11.3)	−50.0	.01
Middle school	43 (40.6)	29 (27.4)	−32.6	.02
High school	92 (86.8)	88 (83.0)	−4.3	.22
Undergraduate	90 (84.9)	88 (83.0)	−2.2	.35
Postgraduate	61 (57.5)	58 (54.7)	−4.9	.34
Program type				
Academic support	93 (87.7)	91 (85.8)	−2.2	.34
Test preparation	60 (56.6)	58 (54.7)	−3.3	.39
Distance learning support	21 (19.8)	42 (39.6)	100	<.001
Mentoring	100 (94.3)	100 (94.3)	0	.50
Psychosocial support	69 (65.1)	71 (67.0)	2.9	.34
Research experience	77 (72.6)	64 (60.4)	−16.9	.03
Shadowing or internships	85 (80.2)	37 (34.9)	−56.5	<.001
Financial support	68 (64.2)	64 (60.4)	−5.9	.29

^a^ *P* values determined using a 2-tailed paired *t* test.

Of the 106 schools that ran pathway programs during the pandemic, 23 schools (21.7%) reported a decrease in funding during the pandemic, including support withheld or diverted to future years. Nine respondents (8.5%) reported an increase in funding, and 71 (67.0%) stated there was no change in funding. Most schools (85 of 106 [80.2%]) received institution/school funding for their pathway programs both before and during the pandemic. Thirty-six of 106 schools (34.0%) received federal funding, 18 of 106 schools (17.0%) received state funding, and 13 of 106 schools (12.3%) received foundation funding both before and during the pandemic. There were no major changes in funding sources after the onset of the pandemic.

### Differences in Programmatic Changes

There were no significant differences in the overall rates of program cancelations or funding changes since the onset of the COVID-19 pandemic across medical school types. However, there were some differences in types of programmatic changes across medical school types. Specifically, private schools (4 of 50 [8.0%]) were significantly more likely than public schools (0 of 56) to start psychosocial support programming during the pandemic (Fischer exact test *P* = .05; χ^2^ *P* = .03). There were no associations found between change in programming and degree type (MD vs DO), region, community-based status, or racial and ethnic minority population–serving status.

### Respondent Perceptions of Pandemic and Future Needs

Most respondents thought the pandemic had a negative effect on engagement and effectiveness of learning in their pathway programs (Table 4). Most respondents reported no effect on participant retention, availability of staff and volunteers, funding, and tracking outcomes. There were mixed responses on the association between the pandemic and participant recruitment, with 35.8% (38 of 106) reporting a negative effect, 22.6% (24 of 106) reporting a positive effect, and 41.5% (44 of 106) reporting no effect. Across all program functions, schools reported more negative or neutral outcomes than positive outcomes.

**Table 4.** Respondent Perceptions of COVID-19 Pandemic Association With Pathway Programs and Implications

Domain	Outcome response, No. (%)		
	Negative	Neutral	Positive
Program function			
Participant			
Recruitment	38 (35.8)	44 (41.5)	24 (22.6)
Engagement	56 (52.8)	25 (23.6)	25 (23.6)
Retention	27 (26.0)	62 (59.6)	15 (14.4)
Social and emotional well-being	57 (55.3)	28 (27.2)	18 (17.5)
Availability			
Staff	26 (24.8)	60 (57.1)	19 (18.1)
Volunteers	38 (36.5)	56 (53.9)	10 (9.6)
Effectiveness of learning	59 (56.7)	27 (26.0)	18 (17.3)
Community engagement	45 (42.9)	42 (40.0)	18 (17.1)
Funding	16 (15.4)	76 (73.1)	12 (11.5)
Tracking outcomes	24 (22.9)	68 (64.8)	13 (12.4)
Opinions on future strategies^a^			
There should be funding for psychosocial support	6 (5.7)	8 (7.6)	92 (86.8)
There should be funding for distance learning	5 (4.7)	8 (7.6)	93 (87.7)
Virtual recruitment is more effective than in-person	44 (41.5)	44 (41.5)	18 (17.0)
Hybrid learning is more effective than in-person	24 (22.6)	39 (36.8)	43 (40.6)
Institutions should deemphasize extracurricular requirements	47 (44.8)	29 (27.6)	29 (27.6)
Funding changes^b^			
How did pathway program funding change as a result of COVID-19?	23 (21.7)	71 (67.0)	9 (8.5)

^a^ For outcome response, the negative response was disagree; neutral, neither agree nor disagree; and positive, agree.

^b^ For outcome response, the negative response was decrease; neutral, none; and positive, increase.

When questioned about future strategies to strengthen pathway programs, most respondents supported increased funding for postpandemic psychosocial support (92 of 106 [86.8%]) and distance learning (93 of 106 [87.7%]). Opinions were varied on whether hybrid learning is better than in-person learning, with 40.6% (43 of 106) of respondents agreeing and 22.6% (24 of 106) disagreeing. In addition, 27.6% (29 of 105) agreed that schools should deemphasize extracurricular activities, such as shadowing, for medical school admissions and 44.8% (47 of 105) disagreed. There was no significant difference in these responses by school type.

Two of the survey questions asked for narrative responses on how the pandemic affected the pathway program in each school. Some general themes that came from these questions included challenges in keeping students engaged in the virtual environment and equity issues regarding broadband internet access, while at the same time recognizing that technology can allow for broader reach. The following 4 sample responses provide additional context to the quantitative findings.

#### Sample Response 1

“Going virtual with some of our pathway programs required us to look at issues of equity regarding internet access and infrastructure issues folks face. Also, we do feel that we lose a bit of the ability for folks to connect in a physical space and create the sense of community we are looking for. On the plus side we did save a bunch of money not having the pathway programs as a residential experience.”

#### Sample Response 2

“Medical schools should de-emphasize extra-curricular requirements in admissions only for those most immediately affected by the pandemic (ie, those who are applying this year and in next year's cycle).”

#### Sample Response 3

“Funding for us is based on a teaching hospital which cut back on ‘elective’ surgeries during COVID-19. Long-term outcomes of this are still being studied. Programming for recruitment went online, as well as elementary, middle, and high school programming. Getting the budgetary requests back to 2019 levels is shaky this coming year.”

#### Sample Response 4

“The COVID-19 pandemic has opened many a Pandora's box as to how ‘stuck’ we seem in ‘old ways’ and it's good to look at ‘disruptive’ ways and those include an emphasis on mental health initiatives.”

## Discussion

The findings of this study suggest that there have been substantial changes to medical school diversity pathway programs since the start of the COVID-19 pandemic. Because programming for younger students appears to be most affected, the outcomes associated with these changes will take decades to be seen. These outcomes may be compounded by broader academic losses among children as a result of pandemic-related school disruptions.^17,18^ Moreover, there is accumulating evidence that these academic losses will not be evenly distributed, but rather will exacerbate preexisting disparities in educational outcomes.^19,20^ Taken together, these findings suggest that pandemic-related changes may have negative outcomes associated with health workforce diversity and equity.

As may be expected, hands-on experiences, such as shadowing and internships, were more likely to be reduced during the pandemic. These decreases may have implications for medical school admissions for students underrepresented in medicine, as shadowing is frequently inquired about during the admissions process.^29^ Nonetheless, most respondents either disagreed or felt neutral about deemphasizing extracurricular activities, such as shadowing and research, in the medical school admissions process. The association between the decrease in shadowing opportunity and applications, admissions, diversity, and professional expectations remains to be seen.^30^

As in the broader health care system, some of the changes noted with the pandemic may have produced unexpected positive outcomes and can be carried forward even when not necessary to comply with public health mandates. For example, schools expressed some positive findings related to distance learning, including reduced costs and expanded reach. Qualitative findings suggest many schools will continue with virtual engagement in some capacity, and there was a high level of agreement among survey respondents that funding for distance learning should be increased. However, given persistent systemic inequities in technological infrastructure from digital redlining,^31^ plans to continue virtual programming must be thoughtfully implemented with an equity lens to consider the results of such choices.

Although most schools did not report substantial decreases in funding for programs, there were nonetheless concerns expressed related to funding and sustainability, particularly in interviews and open-ended questions. Uncertainties around future funding, particularly when funding is related to larger economic concerns in the health care industry, may be a barrier to implementing continued high-quality programming and building trust with community partners.

### Strengths and Limitations

This study has limitations. There were several limitations to the generalizability of these findings. Although purposive and snowball sampling were used for the semistructured interviews and interviews continued until thematic saturation was reached, nonresponse bias and the relatively small sample size may have led to a sample that is not representative of medical schools across the US. However, because the survey was administered to all schools in the US and achieved a relatively high response rate, the quantitative results are likely more generalizable. This high response rate, as well as the similarities in characteristics between respondents and nonrespondents, were strengths of the study. As with most survey studies, responses may have been affected by social desirability bias, particularly because respondents were asked to identify their school to track completion rates; recall bias; and nonresponse bias. This study focused on medical schools, but there are diversity pathway programs within health professions schools of all types. In addition, the findings may be limited by the timeframe during which the study was conducted, as schools had likely only undergone 1 program cycle since the start of the pandemic.

## Conclusions

Future work could examine the association between the pandemic and other health professions school types, additional changes to programming over time, and other factors that may affect the diversity of the student body beyond pathway programs. It may be useful to further characterize the association between the pandemic and funding streams, relations with community partners, persistence of virtual or hybrid models, and the results noted between these changes and program outcomes. Policy makers and medical education leaders may wish to consider the potential negative outcomes of these lost opportunities.

The COVID-19 pandemic continues to have an association with disparate health and educational outcomes of marginalized racial and ethnic groups and communities. The need for a robust physician workforce representative of the diversity of the US is critical to improving health outcomes and eliminating health disparities. Active support for pathway programs and other policy and programmatic opportunities to enhance the diversity of the health professions may be needed to achieve this goal.

## Notes

Supplement. **eAppendix 1.** Key Informant Interview Guide and Questions for School Leaders **eAppendix 2.** Survey Instrument
