# Supplementary material for: Association of the COVID-19 Pandemic With Medical School Diversity Pathway Programs
Source: JAMA Netw Open. 2022 Aug 29;5(8):e2229086. doi: 10.1001/jamanetworkopen.2022.29086 (PMC9425144; doi:10.1001/jamanetworkopen.2022.29086)
Supplement: Supplement. — eAppendix 1. Key Informant Interview Guide and Questions for School Leaders eAppendix 2. Survey Instrument [file jamanetwopen-e2229086-s001.pdf]

Supplementary Online Content

Batra S, Orban J, Raichur S, et al. Association of the COVID-19 pandemic with medical school diversity pathway programs. *JAMA Netw Open*. 2022;5(8):e2229086. doi:10.1001/jamanetworkopen.2022.29086

eAppendix 1. Key Informant Interview Guide and Questions for School Leaders
eAppendix 2. Survey Instrument

This supplementary material has been provided by the authors to give readers additional information about their work.

© 2022 Batra S et al. *JAMA Network Open.*

eAppendix 1: Key Informant Interview Guide and Questions for School Leaders

Introduction to Phone Interview
Hi, My name is Sonal Batra, the principal investigator (or Julie Orban, research scientist) from the Fitzhugh Mullan Institute for Health Workforce Equity at The George Washington University. On the call with me today are several members of my research team (introduce whoever is on).

Before we get started, we wanted to thank you for taking the time to participate on this call. We understand that you may have many other competing priorities, so we really appreciate your willingness to share your thoughts.

I want to briefly review the purpose of this call. Through this interview, we have 2 main aims: First, we want to understand how your school's pipeline program(s) have been impacted by COVID-19. Second, we want to understand if there are methods for sustaining and supporting pipeline programs during the pandemic. Your participation will help inform a broader survey on this topic.

At this time, we would like to ask for your consent to record our conversation so that we may better capture what is being said. The recording is for our review only and will remain confidential—only myself and the other members of the research team will have access to identifiable data. All data collected will be deidentified and reported only in aggregate. Is that okay with you?

As a reminder, this conversation is entirely voluntary and we can stop at any time.

Do you have any questions before we get started? [Answer questions]
Can you please confirm you have received the informed consent document? [Confirms]

QUESTIONS
[Main questions are listed in numerical order below with bulleted probes following each question if necessary.]

Program Characteristics

1. What degrees are offered at your school?
2. What type of pipeline program do you offer students?
    - Are your pipeline program(s) K-12 or undergraduate focused?
    - What ages/academic levels are targeted?
3. How many years have your pipeline program(s) been established?
4. What are the funding sources for your pipeline program?
5. Do you have dedicated institutional support for your program(s)?

© 2022 Batra S et al. *JAMA Network Open.*

- Who provides program support within your school?
- How many people are employed to provide support at either full-time or part-time capacity?

6. What population of students does the program seek to serve?
   - Who is the target population by race/ethnicity of your pipeline program?
   - How do you recruit students to participate in your program?
7. Pre-COVID, what were the features of your pipeline program offered to students?
8. What are your pipeline program goals or objectives?
   - Is increasing the number of underrepresented minorities a part of the mission statement or strategic plan for your school?

Covid-19 Impact

1. How have your pipeline programs been impacted by COVID-19?
   - What types of operational or programmatic characteristics have been impacted?
   - What methods has your school put in place to sustain and support pipeline programs during COVID-19?
   - If the program was suspended, were program funds diverted to other uses?
   - Are there financial impacts?
   - Are there personnel support impacts?
2. How have the number of students participating in your medical school's pipeline programs changed in 2020 compared to 2019?
   - What was your medical school's pipeline program enrollment in 2019?
   - What is your medical school's pipeline program enrollment in 2020, 2021?
   - How many students does your pipeline program(s) typically serve each year?
3. What are the success stories of your pipeline program during COVID-19?
4. What are the challenges your staff face when implementing your program during COVID-19?
   - What programmatic adjustments have been made due to COVID-19?
   - How are you continuing to run your pipeline program?
   - How has your staff addressed the unique needs of your students?
   - Has your school executed any additional activities to supplement the changes in pipeline programs?
   - What factors prevent your pipeline program from succeeding in increasing student enrollment?
   - What would help you and your staff increase pipeline program student enrollment?
5. Do you anticipate any changes in the status to your pipeline programs in 2021?
6. What additional opportunities are there for recruitment, retention, and professional development of URM pursuing medical health careers during COVID-19?
7. How have addressing social/emotional needs for students differed this past year than in previous years?

Recommendations

1. What recommendations do you have to improve the diversity of health professions education?
2. What recommendations do you have to improve pipeline programs and participation?

© 2022 Batra S et al. *JAMA Network Open.*

eAppendix 2: Survey Instrument

Start of Block: Introduction

Thank you so much for taking the time to complete this survey. Your response will contribute to our understanding of the impact of the COVID-19 pandemic on medical school pathway programs. In this research, we are using the term 'pathway program' to describe programs designed to increase the representation of underrepresented minorities and disadvantaged students in the health professions. Pathway programs have also been referred to as pipeline, enrichment, or pre-professional programs.
When completing the survey, please consider ALL your medical school's pathway programs, regardless of funding streams and scopes.

End of Block: Introduction

Start of Block: Section 1: Background

Questions 1 and 2 will only be used to ensure that there are no duplicate responses from a given school AND to correlate results on this survey with externally available data on school characteristics. All data will only be reported in aggregate and no individual schools or roles will be named.

1. What is the name of your medical school?

____

2. What is the job title for your current position?

____

End of Block: Section 1: Background

Start of Block: Section 2: Pathway Program Status

© 2022 Batra S et al. *JAMA Network Open.*

HRSA defines underrepresented minority as an individual from a racial or ethnic group considered inadequately represented in a specific profession relative to the representation of that racial or ethnic group in the general population.

Note: For the purposes of the health professions, we consider individuals from the following racial and ethnic backgrounds underrepresented:

American Indian or Alaska Native

Black or African American

Native Hawaiian or Other Pacific Islander

Hispanic (all races)

---

3. **Prior** to the start of the COVID-19 pandemic (March 2020), was your school sponsoring, running or assisting with any pathway programs targeted to K-12 and/or undergraduate college students who are underrepresented in medicine aimed at encouraging or preparing them to train for careers in the health professions?

- ◯ Yes
- ◯ No
- ◯ Don't know

---

4. **During** the COVID-19 pandemic, has your school been sponsoring, running or assisting with any pathway programs targeted to K-12 and/or undergraduate college students who are underrepresented in medicine aimed at encouraging or preparing them to train for careers in the health professions?

- ◯ Yes
- ◯ No
- ◯ Don't know

© 2022 Batra S et al. *JAMA Network Open.*

5. Did your school cancel any pathway programs in 2020 because of the COVID-19 pandemic?

- ○ Yes
- ○ No
- ○ Don't know

Questions 6 and 7 ask about pathway programs that you ran prior to the pandemic, including those that may have been cancelled, **as well as** pathway programs that you are currently running

6. What grade level(s) do your medical school's pathway program(s) target?
[check all that apply]

	**Prior to the pandemic**	**During the pandemic**
Elementary School (grades K-5)	☐	☐
Middle School (grades 6-8)	☐	☐
High School (grades 9-12)	☐	☐
Undergraduate	☐	☐
Post Undergraduate	☐	☐

© 2022 Batra S et al. *JAMA Network Open.*

7. How are your medical school's pathway program(s) funded? [check all that apply]

	Prior to the pandemic	During the pandemic
Federal Funding (e.g., Health Resources and Services Administration [HRSA], National Institutes of Health)	☐	☐
Foundation Funding (e.g., Robert Wood Johnson Foundation [RWJF], Kaiser Permanente)	☐	☐
State Funding (e.g., Educational Opportunity Fund [EOF])	☐	☐
Institution/School Funding	☐	☐
Other (write-in option)	☐	☐
Other (additional write-in option)	☐	☐

8. Overall how did your medical school's pathway program(s) funding change as a result of the COVID-19 pandemic?

○ Decrease in funding (including funds diverted to other programs or held for subsequent years)

○ Increase in funding

○ No change

○ Don't know

© 2022 Batra S et al. *JAMA Network Open.*

9. What types of programming do you provide in your medical school's pathway program(s)? [check all that apply]

	**Prior to the pandemic**	**During the pandemic**
Academic support (e.g., education and assistance with college application process, writing personal statements, academic enrichment)	☐	☐
Test preparation (e.g., MCAT)	☐	☐
Distancing learning support (e.g., Internet [broadband, WIFI], laptops, web cameras)	☐	☐
Mentoring (e.g., Advising, coaching, counseling)	☐	☐
Psychosocial support (e.g., Personal, social and emotional well-being)	☐	☐
Research experience	☐	☐
Shadowing or internships (e.g., hands-on medical skills, exposure to health professions)	☐	☐
Financial support (e.g., Stipends)	☐	☐
Other (write-in option)	☐	☐

© 2022 Batra S et al. *JAMA Network Open.*

10. What has the impact of the COVID-19 pandemic been on your school's pathway programs in each of the following categories?

	Very negative	Negative	No effect	Positive	Very positive
**Recruitment** of pathway student participants	○	○	○	○	○
**Engagement** of pathway student participants	○	○	○	○	○
**Retention** of pathway student participants	○	○	○	○	○
Social and emotional well-being of pathway student participants	○	○	○	○	○
Availability of staff (full-time and part-time) to support your program(s)	○	○	○	○	○
Availability of volunteers to support your program(s)	○	○	○	○	○
Effectiveness of learning experience for students	○	○	○	○	○
Community/Partner engagement	○	○	○	○	○
Funding	○	○	○	○	○
Ability to track participant outcomes	○	○	○	○	○

© 2022 Batra S et al. *JAMA Network Open.*

Page Break

11. Were there any other changes during the COVID-19 pandemic that had an **unexpected positive or negative impact** on your school's pathway program(s)?

____

____

____

____

____

© 2022 Batra S et al. *JAMA Network Open.*

12. **Post-pandemic**, do you support or oppose the following statements?

	Strongly disagree	Disagree	Neither agree nor disagree	Agree	Strongly agree
There should be funding in grant opportunities to provide psychosocial support (e.g., Personal, social and emotional well-being) to pathway students	○	○	○	○	○
There should be funding in grant opportunities to equip students for distance learning, including access to the internet, access to devices, and training to use the technology	○	○	○	○	○
A virtual/online recruitment process is more effective than in-person recruitment	○	○	○	○	○
Hybrid learning models (a mix of in-person and online instruction) are more effective than fully in-person instruction	○	○	○	○	○
Institutions/schools should de-emphasize extracurricular requirements (e.g., research or shadowing experience) in medical school admissions post COVID-19 pandemic	○	○	○	○	○

© 2022 Batra S et al. *JAMA Network Open.*

13. Please share any additional comments regarding the pandemic's impact on your pathway program(s) below:

____

____

____

____

____

End of Block: Section 2: Pathway Program Status

Start of Block: TY

Thank you for completing the survey. Advancing the survey will automatically submit your responses and you will not have the opportunity to return to your answers. Please take a moment to review your answers before submitting.

End of Block: TY

© 2022 Batra S et al. *JAMA Network Open.*
